# Causes of death and effect of non-cancer-specific death on rates of overall survival in adult classic Hodgkin lymphoma: a populated-based competing risk analysis

**DOI:** 10.1186/s12885-021-08683-x

**Published:** 2021-08-25

**Authors:** Jie Gao, Yingying Chen, Pengqiang Wu, Fujue Wang, Huan Tao, Qianqing Shen, Shuoting Wang, Shuaige Gong, Xue Zhang, Zhencang Zhou, Xianmin Song, Yongqian Jia

**Affiliations:** 1 Department of Hematology, Shanghai General Hospital, Shanghai Jiao Tong University, Shanghai, China; 2 Department of Hematology, West China Hospital, Sichuan University, Chengdu, China; 3 Department of Hematology, The Affiliated Hospital of Southwest Medical University, Luzhou, China; 4 Department of Hematology, The First Affiliated Hospital of University of South China, Hengyang, China; 5 Department of Hematology, The Third Affiliated Hospital of Zunyi Medical University, Guizhou, China

**Keywords:** Classic Hodgkin lymphoma, non-cancer-specific death, competing risk analysis, Prognosis, SEER database

## Abstract

**Background:**

The improved prognosis of classic Hodgkin lymphoma (cHL) has been accompanied by elevated risks of non–cancer-specific death (non-CSD). The aim of this study was to verify the occurrence of non-CSD and its effect on rates of overall survival among adult patients with cHL.

**Methods:**

To ensure sufficient follow-up time, we analyzed retrospective data from patients aged ≥20 years with cHL that was diagnosed between 1983 and 2005 in the Surveillance, Epidemiology, and End Results (SEER) database. Logistic regression was applied to analyze the non-CSD occurrence in relation to all factors. Using Fine-Gray’s method, we calculated the cumulative incidences of CSD and non-CSD. Stacked cumulative incidence plots and ratio of non-CSD to all causes of death were applied to evaluate the effect of non-CSD on rates of overall survival. Finally, we analyzed long-term mortality through Cox proportional hazard regression analysis and competing risk regression analysis to emphasize a more appropriate model of survival for patients with cHL.

**Results:**

Among the 18,518 patients included, there were 3768 cases of CSD (20.3%) and 3217 of non-CSD (17.4%). Older age, earlier period, male sex, unmarried status, mixed cellularity (MC) and lymphocyte-depletion (LD) histological subtype, and patients received radiotherapy (RT) only were associated with more non-CSD according to binary logistic analysis. The cumulative incidence of non-CSD exceeded CSD after approximately 280 months follow-up. The most common causes of non-CSDs were cardiovascular disease, subsequent primary neoplasms, infectious diseases, accidents, and suicide. In a Cox proportional hazards model, patients who were black, unmarried, at an advanced stage or underwent chemotherapy (CT) alone were at greater risk of mortality than were white patients, who were married, at an early stage, and underwent combined modality; these populations were also found to be at greater risk for CSD in a competing risk model, but the risk of non-CSD did not differ significantly according to race and marital status, patients with early-stage disease and who underwent RT only were found to be at higher risk of non-CSD instead.

**Conclusions:**

Lymphoma was the cause of death in most patients who died, but non-CSD was not unusual. Patients with cHL should be monitored closely for signs of cardiovascular disease and malignant tumors. Rates of overall survival of patients were diminished by non-CSD, and a competing risk model was more suitable for establishing the prognosis than was the Cox proportional hazards model.

**Supplementary Information:**

The online version contains supplementary material available at 10.1186/s12885-021-08683-x.

## Background

Hodgkin lymphoma is a rare B-cell malignancy that accounts for about 10% of newly diagnosed lymphomas in the United States [1]. There are two types: nodular lymphocyte-predominant Hodgkin lymphoma and classic Hodgkin lymphoma (cHL). The subclassification cHL, based on abundance of Hodgkin Reed/Sternberg cells, their structure, and histologic features of background infiltration, has four subgroups: nodular sclerosis, mixed cellularity, lymphocyte-rich, and lymphocyte-depletion cHL [2]. The treatment strategies for cHL changed over time, and the treatment plan for early and advanced patients is different. With the improvements in the CT and RT, from the RT alone therapy in the 1980s to the subsequent combined modality treatment, patients with newly diagnosed cHL have a good prognosis, with a cure rate of > 80%; thus, a large number of patients survive. Mortality from causes other than cHL and late medical morbidity is common [3], and studies have revealed an increased risk of death from a subsequent primary neoplasm [4] and cardiovascular disease [5, 6].

In cancer research, overall survival based on the absolute risk of death is considered the most important endpoint to demonstrate whether new treatments can be directly beneficial. The Kaplan-Meier and Cox proportional hazard regression models are the classic techniques in survival analysis, clarifying the association between risk factors and clinical outcomes and predicting the risk of an individual’s clinical outcome through hazard ratios [7, 8], but in none of them are competing causes of death considered. For example, old age, advanced stage, and the lymphocyte-depletion subtype are associated with a relatively poor prognosis, and affected patients with cHL are more prone to CSD [9]. In contrast, young patients and those with early-stage disease have a better prognosis, and the rate of lymphoma-specific mortality is lower; however, these patients may at higher risk for non-CSD. Furthermore, patients treated with CT and/or RT are at risk of developing a secondary malignancy and cardiovascular disease [9]. Thus, overall survival may not be the ideal measure for estimating survival. A competing risk analysis may be more appropriate for outcome analysis of cHL [10, 11].

In previous studies of cHL, overall survival has been assessed with a large population database [12–15], but to our knowledge, no large-scale study of cHL has included competing risk [16]. We conducted a logistic regression of patient data in the SEER database to verify independent factors related to non-CSD rate and performed a competing risk analysis to investigate the effect of non-CSD on rates of overall survival. We tried to define the optimal statistical method by evaluating and comparing the applicability of the Cox proportional hazards regression model and the competing risk regression model.

## Methods

### Data source

We used data from the National Cancer Institute’s SEER 18 registry database (https://seer.cancer.gov/), which represents 28% of the United States population. We used the SEER*Stat software (version 8.3.5) of the National Cancer Institute (available from: https://seer.cancer.gov/seerstat/) to extract data.

### Study information

To ensure sufficient follow-up time, patients aged ≥20 years with cHL that was histologically confirmed between 1983 and 2005 were included in the cohort. Descriptive data were extracted for all patients: age at diagnosis, race, sex, histological type, stage of the Ann Arbor Staging Classification for Hodgkin Lymphoma, information about RT and CT, information about survival, and causes of death. The cancer specific death is due to HL, and non-cancer-specific death is all other cause. Patients for whom any of these data were missing were excluded.

### Statistical analysis

Binary logistics were conducted to investigate the occurrence of non-CSD in relation to all variables. Fine-Gray’s method was used to estimate the cumulative incidence of non-CSD with CSD as a competing risk (and vice versa), and Kaplan-Meier estimation was used to calculate the cumulative incidence of all cancer deaths (ACD). The ratio of non-CSD to ACD and stacked cumulative incidence function plots were applied to estimate the effect of non-CSD on rates of overall survival. The Cox proportional hazards regression model was used to calculate the hazard ratios of all variables for ACD, a subdistribution hazard ratio (SHR) was calculated from the Fine-Gray proportional hazard model (the competing regression model) to predict the association of all variables with CSD and non-CSD. The Cox and Fine-Gray models were assessed with Stata/SE software (version 14.0). The cumulative incidence plots, Kaplan-Meier plots, and forest plots were developed with the cmprsk, survival, and forestplot packages in R software (version 3.6.3). A two-sided *p* value of < 0.05 was considered statistically significant.

## Results

### Patients’ characteristics and causes of deaths

A total of 18,518 eligible patients with cHL were included in this study. Of these patients, 57% were 20 to 39 years of age. The median duration of follow-up was 160 months. Of all the patients, 3768 (20.3%) suffered CSD and 3217 (17.4%) suffered non-CSD. Mortality increased with age and decreased with period. In 1983–1992, patients suffered more non-CSD than CSD (28.1% vs 26.5), and over time, the incidence of CSD gradually exceeded that of non-CSD. More male patients (40.3%) than female patients (34.3%) died. CSD were more common among black patients (25.4%) than among white patients (19.8%). Among the patients with lymphocyte-depletion cHL, CSD accounted for 52.0% of all patients, which is a fairly high proportion. Advanced-stage patients died more often from non-CSD than early-stage patients (18.4% vs 16.8%). Patients who received combined modality had the lowest mortality. Non-CSD were more common among patients who had undergone RT only (27.3%). Detailed information is shown in Table 1. The 10-year estimated probabilities of CSD, non-CSD, and ACD were 17.8, 9.5, and 27.3%, respectively, and the 15-year estimated probabilities were 19.7, 13.8 and 33.5%, respectively; these data were obtained with the Fine-Gray and Kaplan-Meier methods.

**Table 1:** Associations of sociodemographic and clinical characteristics with non-CSD

Variable	No. (%)				Univariate Analysis		Multivariate Analysis	
	Total	Alive	CSD	Non-CSD	OR (95% CI)	***p***-value	OR (95% CI)	***p***-value
**Age, years**								
20–39 (youngest)	10,556	8332 (78.9)	1302 (12.3)	922 (8.7)	Reference		Reference	
40–59 (older)	4734	2758 (58.2)	1011 (21.4)	965 (20.4)	2.68 (2.43–2.95)	< 0.001	3.05 (2.74–3.39)	< 0.001
≥ 60 (oldest)	3228	443 (13.7)	1455 (45.1)	1330 (41.2)	7.32 (6.64–8.07)	< 0.001	8.60 (7.70–9.62)	< 0.001
**Period**								
1983–1992	5022	2282 (45.4)	1331 (26.5)	1409 (28.1)	Reference		Reference	
1993–2000	5743	3621 (63.0)	1142 (19.9)	980 (17.1)	0.53 (0.48–0.58)	< 0.001	0.50 (0.43–0.56)	< 0.001
2001–2005	7753	5640 (72.2)	1295 (16.7)	818 (10.6)	0.31 (0.28–0.34)	< 0.001	0.28 (0.25–0.31)	< 0.001
**Sex**								
Male	9998	5734 (57.4)	2270 (22.7)	1994 (19.9)	Reference		Reference	
Female	8520	5799 (68.0)	1498 (17.6)	1223 (14.4)	0.67 (0.62–0.73)	< 0.001	0.68 (0.62–0.74)	< 0.001
**Race**								
White	16,082	10,070 (62.6)	3180 (19.8)	2832 (17.6)	Reference		Reference	
Black	1694	990 (58.4)	430 (25.4)	274 (16.2)	0.90 (0.79–1.03)	0.139	1.13 (0.97–1.31)	0.117
Others	742	473 (63.7)	158 (21.3)	111 (15.0)	0.82 (0.67–1.01)	0.064	0.91 (0.72–1.14)	0.396
**Marital status**								
Married	9789	6023 (61.6)	1921 (19.6)	1845 (18.8)	Reference		Reference	
Unmarried	8729	5630 (64.5)	1727 (19.8)	1372 (15.7)	0.80 (0.74–0.87)	< 0.001	1.132 (1.04–1.23)	0.005
**Histological type**								
NS	13,562	9362 (69.1)	2284 (16.8)	1916 (14.1)	Reference		Reference	
MC	3804	1658 (43.5)	1117 (29.4)	1029 (27.1)	2.25 (2.07–2.46)	< 0.001	1.19 (1.08–1.31)	< 0.001
LR	729	408 (56.0)	147 (20.1)	174 (23.9)	1.91 (1.60–2.28)	< 0.001	1.09 (0.89–1.32)	0.413
LD	423	105 (24.8)	220 (52.0)	98 (23.2)	1.83 (1.46–2.31)	< 0.001	0.67 (0.52–0.87)	0.002
**Ann Arbor stage**								
Early	11,744	8034 (68.4)	1740 (14.8)	1970 (16.8)	Reference		Reference	
Advanced	6774	3565 (52.6)	1962 (28.9)	1247 (18.5)	1.12 (1.04–1.21)	< 0.001	0.92 (0.85–1.02)	0.393
**Therapy**								
Combined modality	5410	4196 (77.6)	633 (11.7)	581 (10.7)	Reference		Reference	
RT only	2985	1707 (57.2)	461 (15.4)	817 (27.4)	3.13 (2.78–3.52)	< 0.001	1.64 (1.43–1.88)	< 0.001
CT only	8013	4501 (56.1)	2113 (26.4)	1399 (17.5)	1.76 (1.59–1.95)	< 0.001	1.08 (0.97–1.22)	0.175
No/unknown	2110	1129 (53.5)	561 (26.6)	420 (19.9)	2.07 (1.80–2.37)	< 0.001	1.09 (0.94–1.27)	0.241

**Fig. 1:**
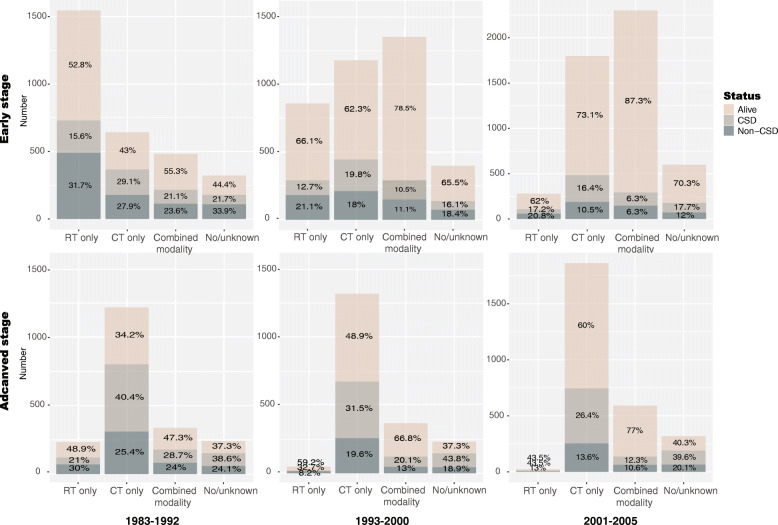
Plots showing early-stage and advanced-stage patients who underwent therapy at different times

The treatment strategies for cHL changed over time, and the treatment plan for early and advanced patients is different. Figure 1 shows that about half of the early-stage patients received only RT in the 1980s, and the mortality rate was high. Non-CSD even exceeded CSD (31.7% vs. 15.6%). The survival rates of early-stage patients receiving CT alone or combination therapy does not exceed 55.3%, while this data in patients at an advanced stage is much poor (Fig. 1). Since 1993, the survival of early and advanced stage patients has significantly improved and the application of CT has increased considerably. By the twenty-first century, the survival rate of early stage cHL patients who received combined modality reached 87.3%, and the survival of advanced-stage patients was also significantly improved, reaching 77%. Detailed information is provided in Fig. 1.

### Association between non-cancer-specific death and patient characteristics

According to the result of multivariate logistic regression analysis, older age, earlier period, male sex, unmarried status, MC and LD histological subtype, and patients who received RT only were associated with more non-CSD (Table 1).

Among all non-CSD, the leading causes of death were cardiovascular disease, subsequent primary neoplasms, infectious diseases, accidents, and suicide (Table 2). Cardiovascular disease accounted for 35.9% of all non-CSD among the oldest patients (aged ≥60), whereas accidents and suicides account for 12.1% of non-CSD in the youngest patients (aged 20 to 39), and the proportion decreased with age. Between 1983 and 1992, the second neoplasm accounted for 21.4% of all non-CSD and decreased with more recent periods, while COPD, cerebrovascular disease, and diabetes mellitus increased with more recent periods. With more advanced Ann Arbor stages, the proportion of deaths from infections also increased (11.5 vs 8.8%). Subsequent primary neoplasms accounted for 22.2% of all non-CSD in patients who received RT only.

**Table 2:** Causes of non-CSD

Variable	Specific deaths/Non-CSD (%)							
	Non-CSD (No.)	Cardiovascular disease	Second neoplasm	Infectious diseases	Accidents/ suicide	COPD	Cerebrovascular	Diabetes Mellitus
**Total**	3217	31.0%	17.8%	8.1%	6.1%	5.1%	3.4%	0.4%
**Age, years**								
20–39 (youngest)	922	28.5%	19.2%	7.5%	12.1%	1.4%	0.5%	1.8%
40–59 (older)	965	26.5%	23.7%	8.2%	5.1%	7.3%	2.6%	1.9%
> 60 (oldest)	1330	35.9%	12.5%	8.6%	2.6%	6.1%	5.9%	2.4%
**Period**								
1983–1992	1409	31.1%	21.4%	9.9%	5.7%	4.8%	2.7%	1.6%
1993–2000	980	31.6%	17.6%	10.7%	6.2%	5.3%	2.8%	2.3%
2001–2005	818	30.2%	15.2%	8.8%	7.0%	5.4%	3.6%	2.5%
**Stage**								
Early stage	1970	31.2%	19.5%	8.8%	5.9%	5.5%	3.6%	2.2%
Advanced stage	1247	31.0%	18.0%	11.5%	6.7%	4.5%	3.1%	1.9%
**Therapy**								
Combined modality	581	33.6%	20.7%	7.1%	7.4%	4.5%	2.2%	1.9%
RT only	817	31.9%	22.2%	9.2%	4.7%	4.5%	3.8%	1.8%
CT only	1399	29.8%	16.4%	11.7%	6.7%	5.6%	3.1%	1.9%
No/unknown	420	31.7%	15.0%	9.3%	6.0%	5.2%	5.0%	3.3%

*CSD* cancer-specific death, *COPD* chronic obstructive pulmonary disease

### The cumulative incidence of non–cancer-specific and cancer-specific death using competing risk analysis

**Fig. 2:**
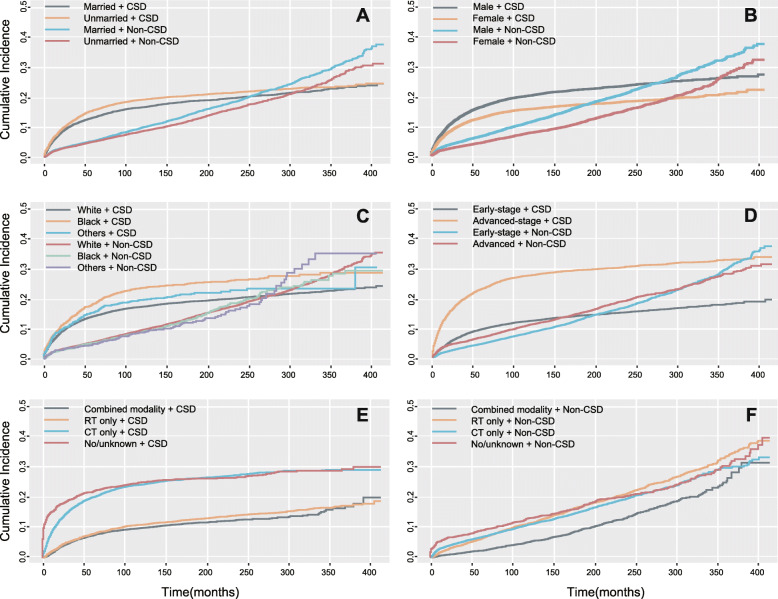
Cumulative incidence of cancer-specific death (CSD) and non-CSD according to characteristics: **A** Marital status; **B** sex; **C** Race; **D** Stage; **E** and **F** Therapy. The curves for deaths of specific causes were generated by Fine-Gray’s method

**Fig. 3:**
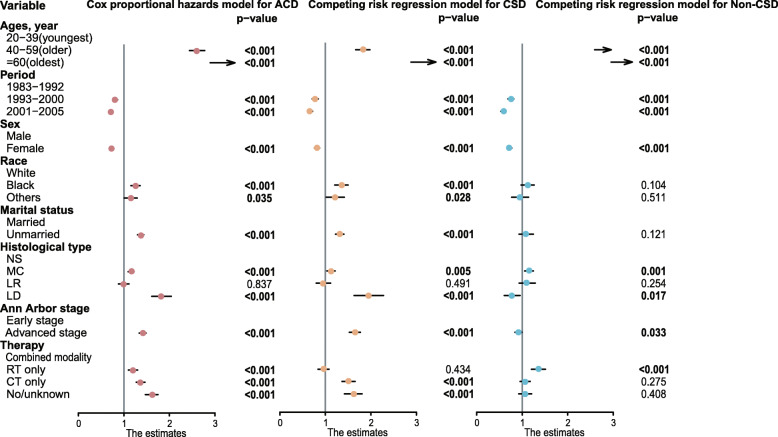
Forest plots showing different results of multivariable analyses for all cancer deaths (ACD), based on the Cox proportional hazards model, and of the multivariable analyses for cancer-specific death (CSD) and non-CSD, based on the competing risk regression model, in patients with classic Hodgkin lymphoma in the Surveillance, Epidemiology, and End Results database. HR, SHR and 95%CI value was listed in Supplementary Tables 1, 2 and 3. HR, hazard ratio; CI, confidence interval; SHR, subdistribution hazard ratio; NS, nodular sclerosis; MC, mixed cellularity; LR, lymphocyte-rich; LD, lymphocyte-depletion

In Fine-Gray’s analysis, results showed that CSD was more common among patients aged 40 to 59 and the oldest patients than in the youngest patients, with SHRs of 1.855 (95% CI: 1.710 to 2.011; *P* < 0.001) and 4.875 (95% CI: 4.521 to 5.265; *P* < 0.001), respectively, as was non-CSD, with SHRs of 2.754 (95% CI: 2.522 to 3.007; *P* < 0.001) and 6.487 (95% CI: 5.967 to 7.053; *P* < 0.001), respectively (Fig. 2A). In 1983–1992, the cumulative incidence of CSD and non-CSD was the highest and gradually decreased with time and treatment changes. Unmarried patients had higher cumulative incidence of CSD (SHR: 1.103; 95% CI: 1.035 to 1.176; *P* = 0.003) and lower cumulative incidence of non-CSD (SHR: 0.844; 95% CI: 0.787 to 0.904; *P* < 0.001) than married patients (Fig. 2A). Female patients had lower cumulative incidence of CSD (SHR: 0.755; 95% CI: 0.707 to 0.806; *P* < 0.001) and non-CSD (SHR: 0.702; 95% CI: 0.654 to 0.753; *P* < 0.001) than did male patients (Fig. 2B). Patients with MC, LR, and LD cHL were at higher cumulative incidence for CSD and non-CSD than patients with the NS subtype. Black patients had higher cumulative incidence of CSD (SHR: 1.343; 95% CI: 1.125 to 1.485; *P* < 0.001) than did white patients, but the cumulative incidences of non-CSD were not significantly different (SHR: 0.962; 95% CI: 0.850 to 1.089; *P* = 0.542; Fig. 2C). Advanced-stage patients suffered higher cumulative incidence of CSD (SHR: 2.263; 95% CI: 2.123 to 2.412; *P* < 0.001) than patients with early-stage disease as well as higher cumulative incidence of non-CSD (SHR: 1.098; 95% CI: 1.023–1.179; *P* = 0.010) (Fig. 2D). Compared with patients who received combined modality, patients who received RT only, CT only, or no therapy suffered more CSD (Fig. 2E) and non-CSD (Fig. 2F). Detailed values are listed in Supplementary Tables 1 and 2. The univariable (Supplementary Table 1) and multivariable analyses (Fig. 3) showed that age, period, sex, marital status, histological subtype, Ann Arbor stage, and treatment strategies were independently predictive of the cumulative incidence of non-CSD.

### Survival analysis: comparison of cox proportion hazards regression model and competing risk regression model

The hazard ratios are illustrated in Fig. 3. Eight covariables—age, period, sex, race, marital status, histological type, Ann Arbor stage, treatment strategies—were associated with the ACD rate in a Cox proportional hazards model. The oldest patients showed the worst prognosis; the hazard ratio for ACD was 9.539 (95% CI: 8.953 to 10.163; *P* < 0.001). The competing risk regression model for CSD yielded similar outcomes (Fig. 3). The oldest patients had highest risks of both CSD (SHR: 4.347; 95% CI: 4.000 to 4.725; *P* < 0.001) and non-CSD (SHR: 7.073; 95% CI: 6.449 to 7.757; *P* < 0.001). According to the competing risk model, unmarried patients had a higher CSD rate (SHR: 1.311; 95% CI: 1.224 to 1.405; *P* < 0.001) than did married patients, but the non-CSD rates did not differ (SHR: 1.054; 95% CI: 0.932 to 1.244; *P* = 0.121). Black patients with cHL were at greater risk of CSD than were white patients (SHR: 1.344; 95% CI: 1.207 to 1.496; *P* < 0.001); however, the non-CSD rates did not differ significantly (SHR: 1.111; 95% CI: 0.978 to 1.261; *P* = 0.104). In comparison with patients who had early-stage disease, patients at an advanced stage had higher rates of ACD and CSD but lower risk of non-CSD in the competing risk model (SHR: 0.915; 95% CI: 0.843 to 0.993; *P* = 0.033). Patients who received RT only, CT only, or who did not receive therapy were at higher risk of ACD than those who received combined modality. The competing risk regression model for CSD yielded similar outcomes, while patients who underwent RT alone suffered higher risk of non-CSD (SHR: 1.349; 95% CI: 1.206 to 1.511; *P* < 0.001). Detailed information about the univariable and multivariable analyses for CSD and ACD according to the competing risk regression model and the Cox proportional hazards regression model are shown in Supplementary Tables 2 and 3.

### Effect of non–cancer-specific death on rates of overall survival

**Fig. 4:**
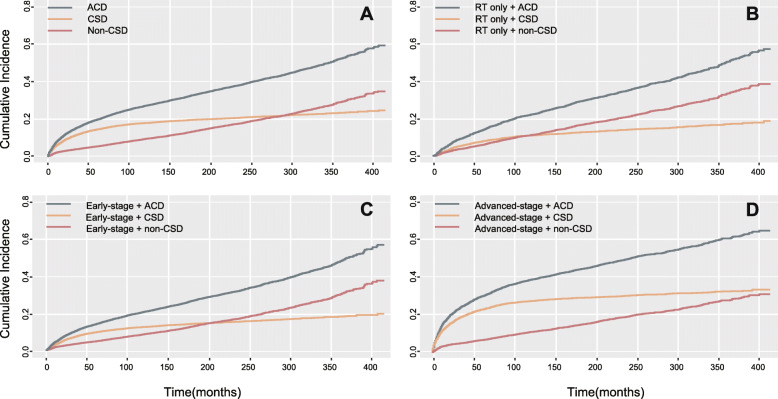
Stacked cumulative incidence plots based on the Kaplan-Meier method. **A** For all patients, the risk of non–cancer-specific death (non-CSD) exceeded that of CSD approximately 280 months after diagnosis. **B** In patients who underwent RT only, non-CSD gradually increased until it exceeded CSD at approximately 120 months. **C** In patients with early-stage disease, non-CSD gradually increased until it exceeded CSD at approximately 200 months. **D** In patients with advanced-stage disease, the risk of CSD always exceeded the risk of non-CSD

Table 3 shows Fine-Gray and Kaplan-Meier estimates of the probability of non-CSD and ACD occurring within 10 and 15 years. Stacked cumulative incidence function plots showed that the cumulative incidence of non-CSD exceeded that of CSD after a follow-up of approximately 280 months (Fig. 4A). After 10 years of follow-up, the ratio of non-CSD to ACD in patients with patients who received RT only was higher than 0.5. Figure 4B outlines the cumulative incidence of non-CSD exceeded CSD after 120 months of follow-up for patients who received RT alone. The ratio of non-CSD to ACD, however, was consistently below 0.3 in the subgroups with poor survival, such as patients with LD cHL. Rates of non-CSD in patients with early-stage disease gradually increased over time until it exceeded CSD at approximately 200 months (Fig. 4C). In the patients with advanced stage disease, the incidence of CSD always exceeded that of non-CSD (Fig. 4D).

**Table 3:** Effect of non-CSD on rates of overall survival

Variable		At 10 years (%)			At 15 years (%)	
	non-CSD (***n*** = 1754)	ACD (***n*** = 5055)	non-CSD/ACD	non-CSD (***n*** = 2422)	ACD (***n*** = 5909)	non-CSD/ACD
**Total**	9.5%	27.3%	0.35	13.8%	33.5%	0.41
**Ages, year**						
20–39 (Youngest)	3.1%	13.3%	0.23	5.1%	16.7%	0.31
40–59 (Older)	9.9%	27.8%	0.36	16.7%	37.3%	0.45
≥ 60 (Oldest)	29.8%	72.5%	0.41	38.2%	83%	0.46
**Period**						
1983–1992	11.4%	32.6%	0.35	15.6%	39.1%	0.40
1993–2000	9.4%	26.9%	0.35	13.3%	32.3%	0.41
2001–2005	8.5%	24.6%	0.35	13.3%	30.7%	0.43
**Sex**						
Male	11.1%	31%	0.36	16.3%	38.2%	0.43
Female	7.6%	23%	0.33	10.8%	27.8%	0.39
**Race**						
White	9.6%	26.8%	0.36	13.9%	32.9%	0.42
Black	9%	32.4%	0.28	13.4%	38.4%	0.35
Others	8.5%	27.8%	0.31	12.2%	33.7%	0.36
**Marital status**						
Married	10.1%	26.8%	0.38	14.8%	33.6%	0.44
Unmarried	8.8%	27.9%	0.32	12.7%	33.4%	0.38
**Histological type**						
NS	7.2%	21.9%	0.33	10.9%	27.2%	0.40
MC	15.9%	41.9%	0.38	21.9%	50.4%	0.43
LR	13%	28.8%	0.45	20.2%	39.1%	0.52
LD	16.5%	65.9%	0.25	20.6%	72.0%	0.29
**Stage**						
Early stage	8.7%	21.4%	0.41	13.0%	27.2%	0.48
Advanced stage	11.2%	38.7%	0.29	15.1%	44.3%	0.34
**Therapy**						
Combined modality	5.2%	15.1%	0.34	9.1%	20.6%	0.44
RT only	11.3%	22.2%	0.51	16.7%	29.3%	0.57
CT only	10.9%	35.1%	0.31	15.1%	41.3%	0.37
No/unknown	13.0%	37.9%	0.34	16.5%	42.6%	0.39

*CSD* cancer-specific death, *ACD* all causes of deaths, *NS* nodular sclerosis, *MC* mixed cellularity, *LR* lymphocyte-rich, *LD* lymphocyte-depletion

## Discussion

Advances in the understanding of lymphoma and improvements in CT and RT have improved survival at various stages of cHL [15]. The relative rate of 5-year survival in patients with newly diagnosed cHL at ages 20 to 64 years was 89.9%; these patients were more likely to die of complications associated with long-term treatment rather than of the lymphoma itself. Aleman et al. reported that 22 years after treatment of adult patients, the risk of death from other causes exceeded the risk of death from Hodgkin lymphoma itself [4]. The most common causes of death for survivors other than Hodgkin lymphoma included a second primary malignant tumor, cardiovascular diseases, and infections [17]. Such a high incidence of non-CSD significantly affects rates of survival and obfuscates the actual rate of long-term mortality caused by cHL. Thus, a competing risk regression model may be more appropriate for outcome analysis. Also, models should account for late mortality, and clinicians must closely monitor patients for signs of possible cardiovascular disease and malignant tumors in patients who may be susceptible to non-CSD.

We found that the cumulative incidence of non-CSD exceeded that of CSD after a follow-up of approximately 280 months (Fig. 4A), which is consistent with reports in the literature [4]. Among most patients with poor prognostic factors such as advanced stage or poor histological type, short-term mortality results from primary lymphoma more often than does non-CSD, as showed in Fig. 4D. The stacked cumulative incidence curve shows that the risk of CSD in patients with advanced stage disease always exceeds the risk of non-CSD. In contrast, patients with early-stage disease have a lower risk of CSD and a higher risk of non-CSD, and the non-CSD/ACD ratio gradually increases over time. The stacked cumulative incidence curve also showed that the risk of non-CSD exceeded the risk of CSD after approximately 200 months (Fig. 4C).

To verify independent factors associated with non-CSD, we used a logistic regression analysis and found that factors such as older age, earlier period, male sex, unmarried status, MC and LD histological subtype, and patients who received RT only were more associated with non-CSD than with CSD. The covariables obtained in the Cox model that have an influence on the survival time of patients with cHL are the same as those identified in the Fine-Gray model, including age, period, sex, race, marital status, histological type, Ann Arbor stage, and treatment (Fig. 3). Age is obviously the most significant determinant affecting survival outcomes. According to the Cox proportional hazards model, the hazard ratio of ACD in oldest patients in relation to youngest patients is 9.539 (*P* < 0.001), and the oldest patients have higher risks of both CSD (SHR: 4.347; *P* < 0.001) and non-CSD (SHR: 7.073; *P* < 0.001) than do youngest patients, according to a competing risk model; therefore, non-CSD has a greater effect on survival in the oldest patients. A competing risk regression model could correct for the overestimation of overall survival. The causes of death were slightly different among different age groups; accident and suicide accounted for 12.1% of non-CSD among the youngest patients, which may be related to greater social activities and social pressure in this age group.

In comparison with unmarried patients, married patients had better survival outcomes with various tumors, including Hodgkin lymphoma [18], Married people may get better hospital treatment than unmarried people, and a partner can provide emotional comfort and financial support [19]. To confirm this hypothesis, we further included patients diagnosed after 2007 in the SEER database and found that married patients have higher insurance rates than unmarried patients (94.8 vs 89.7%) (data not shown). In our study, the Cox proportional hazards model demonstrated that unmarried patients had worse overall survival than did married patients; in the competing risk model, the unmarried patients also had a higher rate of CSD (*P* < 0.001), but the non-CSD rate was not different from that of married patients (*P* = 0.121). Perhaps the main benefit of marriage for patients with cHL is to reduce the mortality from CSD.

Worse overall survival has also been found among patients who were black; rates of death were higher among nonwhite children and adolescents with cHL than among their white counterparts [20]. In our study, black patients had a higher rate of mortality than did white patients, according to the Cox proportional hazards model (*P* < 0.001), and the CSD rate was also higher in the competing risk model (*P* < 0.001), but rates of non-CSD did not differ between races (*P* = 0.104). We also conducted insurance analysis in patients diagnosed after 2007 and found that the insurance coverage rate for whites was 92.4% (7743/8377) whereas that for blacks was 88.4% (1043/1181), which was slightly lower than that for whites. With insurance coverage, the ACD and CSD rates in black patients were higher than those in white patients, but the non-CSD rates were very close (data now shown). Perhaps the racial advantage of white people is reflected only in the reduction of CSD.

RT and CT are the mainstay of treatment for primary cHL [21]. In the 1980s, RT alone in early-stage patients showed high chances of cure [22], and extended field radiotherapy (EFRT) was considered the standard treatment for early-stage cHL patients. However, due to the high recurrence rate and long-term complications, EFRT involving adjacent lymph node areas was no longer used. Subsequent prospective randomized studies established combined modality treatment, including ABVD (doxorubicin, bleomycin, vinblastine, and dacarbazine) regimens and involved-field radiotherapy (IFRT), as the standard therapy for early-stage patients [23–26]. This is also reflected in our results. As is shown in Fig. 1, about half of the early-stage patients received only RT in the 1980s, the mortality rate was high, and non-CSD even exceeded CSD. In the whole cohort, subsequent primary neoplasms accounted for 22.2% of all non-CSD in patients who received RT only. Additionally, multiple competing risk analysis avoiding the interference effect of other factors showed that patients who underwent RT alone suffered higher risk of non-CSD. The survival rate of patients with advanced cHL is less than 5% without treatment or single-agent CT [15]. Multi-center studies show that ABVD is superior to MOPP (mechlorethamine, vincristine, procarbazine, and prednisone) in overall survival and progression-free survival, and long-term follow-up confirms this view [27–29]. As we can see in our results, patients’ survival time improved as treatment regimens progressed. By the twenty-first century, the survival rate of early-stage cHL patients who received combined modality reached 87.3%, and the survival of advanced-stage patients has also significantly improved, reaching 77%.

The goal of future research on limited-stage Hodgkin lymphoma is to simultaneously maintain or increase long-term cure rates while reducing toxicity [30–32]. Intensified screening should be performed after the end of treatment. At each follow-up examination, patient should be queried about symptoms that may indicate treatment of sequelae, and cardiograms, echocardiography examinations and pulmonary function testing should be conducted in patients who received CT or mediastinal RT [9].

To the best of our knowledge, this study is the first in which a competing risk analysis model was used to assess independent prognostic factors in cHL in a large population of patients. This population was monitored for a sufficiently long time, and the results were reliable. However, our research still has limitations: Patient information in the SEER database is limited; some information, including specific CT regimens, lymphoma invasion sites, and genetic testing, have not been recorded; and there exists coding errors. Second, the nature of retrospective study does impose statistical bias and it would be impossible to eliminate residual confounding.

In summary, non-CSD has a great effect on rates of survival in a long follow-up of patients with cHL. Patients should be monitored closely for signs of possible cardiovascular disease and malignancies, and early intervention is very important. Moreover, a competing risk analysis may be more appropriate for outcome analysis of cHL.

## Supplementary Information

**Additional file 1: Supplementary Table 1.** Univariable and multivariable analyses of non-CSD: A competing risk regression model. **Supplementary Table 2.** Univariable and multivariable analyses of CSD: A competing risk regression model. **Supplementary Table 3.** Univariable and multivariable analyses of ACD in patients with cHL in the Surveillance, Epidemiology, and End Results database: Cox proportional hazards model.

## Data Availability

The datasets analyzed for this study can be found in the Surveillance, Epidemiology, and End Results (SEER) database. https://seer.cancer.gov/.

## Abbreviations

cHL: Classic Hodgkin lymphoma
CSD: Cancer-specific death
Non-CSD: Non-cancer-specific death
SEER: Surveillance, Epidemiology, and End Results
CT: Chemotherapy
RT: Radiotherapy
NS: Nodular sclerosis
MC: Mixed cellularity
LR: Lymphocyte-rich
LD: Lymphocyte-depletion
HR: Hazard ratio
SHR: Subdistribution hazard ratio
EFRT: Extended field radiotherapy
IFRT: Involved-field radiotherapy
ABVD: Doxorubicin, bleomycin, vinblastine, and dacarbazine
MOPP: Mechlorethamine, vincristine, procarbazine, and prednisone

## References

[1] Shanbhag S, Ambinder RF (2018). Hodgkin lymphoma: a review and update on recent progress. CA Cancer J Clin.

[2] Sehn LH (2018). Introduction to a review series on Hodgkin lymphoma: change is here. Blood.

[3] Kiserud CE, Loge JH, Fossa A, Holte H, Cvancarova M, Fossa SD (2010). Mortality is persistently increased in Hodgkin's lymphoma survivors. Eur J Cancer.

[4] Aleman BM, van den Belt-Dusebout AW, Klokman WJ, Van't Veer MB, Bartelink H, van Leeuwen FE (2003). Long-term cause-specific mortality of patients treated for Hodgkin's disease. J Clin Oncol.

[5] Galper SL, Yu JB, Mauch PM, Strasser JF, Silver B, Lacasce A, Marcus KJ, Stevenson MA, Chen MH, Ng AK (2011). Clinically significant cardiac disease in patients with Hodgkin lymphoma treated with mediastinal irradiation. Blood.

[6] Myrehaug S, Pintilie M, Tsang R, Mackenzie R, Crump M, Chen Z, Sun A, Hodgson DC (2008). Cardiac morbidity following modern treatment for Hodgkin lymphoma: supra-additive cardiotoxicity of doxorubicin and radiation therapy. Leuk Lymphoma.

[7] Jhawar SR, Rivera-Nunez Z, Drachtman R, Cole PD, Hoppe BS, Parikh RR (2019). Association of Combined Modality Therapy vs chemotherapy alone with overall survival in early-stage pediatric Hodgkin lymphoma. JAMA Oncol.

[8] Hertzberg MS, Crombie C, Benson W, Taper J, Gottlieb D, Bradstock KF (2006). Outpatient fractionated ifosfamide, carboplatin and etoposide as salvage therapy in relapsed and refractory non-Hodgkin's and Hodgkin's lymphoma. Ann Oncol.

[9] Bröckelmann PJ, Eichenauer DA, Jakob T, Follmann M, Engert A, Skoetz N (2018). Hodgkin lymphoma in adults. Dtsch Arztebl Int.

[10] Wu J, Man D, Wang K, Li L (2019). Impact of nonappendiceal cancer-specific death on overall survival: a competing risk analysis. Future Oncol.

[11] Fu J, Wu L, Jiang M, Li D, Jiang T, Fu W, Wang L, Du J (2017). Real-world impact of non-breast cancer-specific death on overall survival in resectable breast cancer. Cancer.

[12] Ebied A, Thanh Huan V, Makram OM, Sang TK, Ghorab M, Ngo HT, Iraqi A, Kamel MG, Dang TN, Vuong NL, Hirayama K, Huy NT (2018). The role of primary lymph node sites in survival and mortality prediction in Hodgkin lymphoma: a SEER population-based retrospective study. Cancer Med.

[13] Zhang Y, Zhang J, Zeng H, Zhou XH, Zhou HB (2017). Nomograms for predicting the overall and cancer-specific survival of patients with classical Hodgkin lymphoma: a SEER-based study. Oncotarget.

[14] Gerber NK, Atoria CL, Elkin EB, Yahalom J (2015). Characteristics and outcomes of patients with nodular lymphocyte-predominant Hodgkin lymphoma versus those with classical Hodgkin lymphoma: a population-based analysis. Int J Radiat Oncol Biol Phys.

[15] Koshy M, Fairchild A, Son CH, Mahmood U (2016). Improved survival time trends in Hodgkin's lymphoma. Cancer Med.

[16] Provencio M, Millan I, Espana P, Sanchez AC, Sanchez JJ, Cantos B, Vargas JA, Bellas C, Garcia V, Sabin P (2008). Analysis of competing risks of causes of death and their variation over different time periods in Hodgkin's disease. Clin Cancer Res.

[17] Matasar MJ, Ford JS, Riedel ER, Salz T, Oeffinger KC, Straus DJ. Late morbidity and mortality in patients with Hodgkin's lymphoma treated during adulthood. J Natl Cancer Inst. 2015;107(4):djv018. 10.1093/jnci/djv018. PMC6281032 25717170

[18] Aizer AA, Chen MH, McCarthy EP, Mendu ML, Koo S, Wilhite TJ, Graham PL, Choueiri TK, Hoffman KE, Martin NE (2013). Marital status and survival in patients with cancer. J Clin Oncol.

[19] Wang F, Xie X, Yang X, Jiang G, Gu J (2017). The influence of marital status on the survival of patients with Hodgkin lymphoma. Oncotarget.

[20] Kahn JM, Kelly KM, Pei Q, Bush R, Friedman DL, Keller FG, Bhatia S, Henderson TO, Schwartz CL, Castellino SM (2019). Survival by race and ethnicity in pediatric and adolescent patients with Hodgkin lymphoma: a Children's oncology group study. J Clin Oncol.

[21] Mottok A, Steidl C (2018). Biology of classical Hodgkin lymphoma: implications for prognosis and novel therapies. Blood.

[22] Mauch PM, Weinstein H, Botnick L, Belli J, Cassady JR (1983). An evaluation of long-term survival and treatment complications in children with Hodgkin's disease. Cancer.

[23] Press OW, LeBlanc M, Lichter AS, Grogan TM, Unger JM, Wasserman TH, Gaynor ER, Peterson BA, Miller TP, Fisher RI (2001). Phase III randomized intergroup trial of subtotal lymphoid irradiation versus doxorubicin, vinblastine, and subtotal lymphoid irradiation for stage IA to IIA Hodgkin's disease. J Clin Oncol.

[24] Engert A, Schiller P, Josting A, Herrmann R, Koch P, Sieber M, Boissevain F, De Wit M, Mezger J, Duhmke E (2003). Involved-field radiotherapy is equally effective and less toxic compared with extended-field radiotherapy after four cycles of chemotherapy in patients with early-stage unfavorable Hodgkin's lymphoma: results of the HD8 trial of the German Hodgkin's lymphoma study group. J Clin Oncol.

[25] Noordijk EM, Carde P, Dupouy N, Hagenbeek A, Krol AD, Kluin-Nelemans JC, Tirelli U, Monconduit M, Thomas J, Eghbali H (2006). Combined-modality therapy for clinical stage I or II Hodgkin's lymphoma: long-term results of the European Organisation for Research and Treatment of Cancer H7 randomized controlled trials. J Clin Oncol.

[26] Blank O, von Tresckow B, Monsef I, Specht L, Engert A, Skoetz N (2017). Chemotherapy alone versus chemotherapy plus radiotherapy for adults with early stage Hodgkin lymphoma. Cochrane Database Syst Rev.

[27] Canellos GP, Anderson JR, Propert KJ, Nissen N, Cooper MR, Henderson ES, Green MR, Gottlieb A, Peterson BA (1992). Chemotherapy of advanced Hodgkin's disease with MOPP, ABVD, or MOPP alternating with ABVD. N Engl J Med.

[28] Anselmo AP, Cartoni C, Bellantuono P, Maurizi-Enrici R, Aboulkair N, Ermini M (1990). Risk of infertility in patients with Hodgkin's disease treated with ABVD vs MOPP vs ABVD/MOPP. Haematologica.

[29] Duggan DB, Petroni GR, Johnson JL, Glick JH, Fisher RI, Connors JM, Canellos GP, Peterson BA (2003). Randomized comparison of ABVD and MOPP/ABV hybrid for the treatment of advanced Hodgkin's disease: report of an intergroup trial. J Clin Oncol.

[30] Brockelmann PJ, Sasse S, Engert A (2018). Balancing risk and benefit in early-stage classical Hodgkin lymphoma. Blood.

[31] Longley J, Johnson PWM (2019). Current treatment paradigms for advanced stage Hodgkin lymphoma. Br J Haematol.

[32] Lim SH, Johnson PWM (2018). Optimizing therapy in advanced-stage Hodgkin lymphoma. Blood.

