# Supplementary material for: Causes of death and effect of non-cancer-specific death on rates of overall survival in adult classic Hodgkin lymphoma: a populated-based competing risk analysis
Source: BMC Cancer. 2021 Aug 25;21:955. doi: 10.1186/s12885-021-08683-x (PMC8390285; doi:10.1186/s12885-021-08683-x)
Supplement: Supplementary file 1 — Additional file 1: Supplementary Table 1. Univariable and multivariable analyses of non-CSD: A competing risk regression model. Supplementary Table 2. Univariable and multivariable analyses of CSD: A competing risk regression model. Supplementary Table 3. Univariable and multivariable analyses of ACD in patients with cHL in the Surveillance, Epidemiology, and End Results database: Cox proportional hazards model. [file 12885_2021_8683_MOESM1_ESM.docx]

Causes of Death and Effect of Non-Cancer-Specific Death on Rates of Overall Survival in Adult Classic Hodgkin Lymphoma: A Populated-Based Competing Risk Analysis

Jie Gao^1^, Yingying Chen^1^, Pengqiang Wu^1,2^, Fujue Wang^1,3^, Huan Tao^1^, Qianqing Shen^1^, Shuoting Wang^1^, Shuaige Gong^1^, Xue Zhang^1^, Zhencang Zhou^1,4^, Yongqian Jia^1*^

^1^Department of Hematology, West China Hospital, Sichuan University, Chengdu, China.

^2^Department of Hematology, The Affiliated Hospital of Southwest Medical University, Luzhou, China.

^3^Department of Hematology, The First Affiliated Hospital of University of South China, Hengyang, China.

^4^Department of Hematology, The Third Affiliated Hospital of Zunyi Medical University, Guizhou, China.

*** Correspondence:**Yongqian Jia
jia_yq@scu.edu.cn

# Supplementary Tables

**Supplementary Table 1.** Univariable and multivariable analyses of non-CSD: A competing risk regression model.

| **Variable** | **Univariate analysis** | |  | **Multivariate analysis** | |
| --- | --- | --- | --- | --- | --- |
|  | **SHR (95%CI)** | **p-value** |  | **SHR(95%CI)** | **p-value** |
| **Ages, year** |  |  |  |  |  |
| 20-39 (youngest) | References |  |  | References |  |
| 40-59 (older) | 2.754 (2.522-3.007) | <0.001 |  | 2.892(2.6378-3.169) | <0.001 |
| ≥60 (oldest) | 6.487(5.967-7.053) | <0.001 |  | 7.073(6.449-7.757) | <0.001 |
| **Period** |  |  |  |  |  |
| 1983-1992 |  |  |  |  |  |
| 1993-2000 | 0.797(0.735-0.866) | <0.001 |  | 0.749(0.689-0.816) | <0.001 |
| 2001-2005 | 0.676(0.619-0.738) | <0.001 |  | 0.586(0.534-0.644) | <0.001 |
| **Sex** |  |  |  |  |  |
| Male | References |  |  | References |  |
| Female | 0.702(0.654-0.753) | <0.001 |  | 0.718(0.668-0.772) | <0.001 |
| **Race** |  |  |  |  |  |
| White | References |  |  | References |  |
| Black | 0.962(0.850-1.089) | 0.542 |  | 1.111(0.978-1.261) | 0.104 |
| Others | 0.941 (0.784-1.136) | 0.528 |  | 0.936(0.770-1.139) | 0.511 |
| **Marital status** |  |  |  |  |  |
| Married | References |  |  | References |  |
| Unmarried | 0.844(0.787-0.904) | <0.001 |  | 1.054(0.932-1.244) | 0.121 |
| **Histological type** |  |  |  |  |  |
| NS | References |  |  | References |  |
| MC | 1.982(1.837-2.138) | <0.001 |  | 1.146(1.057-1.244) | 0.001 |
| LR | 1.749(1.500-2.040) | <0.001 |  | 1.097(0.936-1.286) | 0.254 |
| LD | 1.703(1.381-2.100) | <0.001 |  | 0.761(0.608-0.953) | 0.017 |
| **Ann Arbor stage** |  |  |  |  |  |
| Early stage | References |  |  | References |  |
| Advanced stage | 1.098(1.023-1.179) | 0.010 |  | 0.915(0.843-0.993) | 0.033 |
| **Therapy** |  |  |  |  |  |
| Combined modality | References |  |  | References |  |
| RT only | 1.883(1.693-2.095) | <0.001 |  | 1.349(1.206-1.511) | <0.001 |
| CT only | 1.601(1.455-1.763) | <0.001 |  | 1.059(0.956-1.174) | 0.275 |
| No/unknown | 1.784(1.573-2.024) | <0.001 |  | 1.057(0.926-1.206) | 0.408 |

SHR, subdistribution hazard ratio; NS, nodular sclerosis; MC, mixed cellularity; LR, lymphocyte-rich; LD, lymphocyte-depletion; RT, radiotherapy; CT, chemotherapy.

**Supplementary Table 2.** Univariable and multivariable analyses of CSD: A competing risk regression model

| **Variable** | **Univariate analysis** | | |  | **Multivariate analysis** | | |
| --- | --- | --- | --- | --- | --- | --- | --- |
|  | | **SHR (95%CI)** | **p-value** |  | **SHR(95%CI)** | **p-value** |  |
| **Ages, year** | |  |  |  |  |  |  |
| 20-39(youngest) | | References |  |  | References |  |  |
| 40-59 (older) | | 1.855(1.710-2.011) | <0.001 |  | 1.815(1.667-1.977) | <0.001 |  |
| ≥60 (oldest) | | 4.875(4.521-5.256) | <0.001 |  | 4.347(4.000-4.725) | <0.001 |  |
| **Period** | |  |  |  |  |  |  |
| 1983-1992 | |  |  |  |  |  |  |
| 1993-2000 | | 0.788(0.728-0.853) | <0.001 |  | 0.774(0.711-0.842) | <0.001 |  |
| 2001-2005 | | 0.712(0.658-0.769) | <0.001 |  | 0.659(0.605-0.718) | <0.001 |  |
| **Sex** | |  |  |  |  |  |  |
| Male | | References |  |  | References |  |  |
| Female | | 0.755(0.707-0.806) | <0.001 |  | 0.814(0.760-0.872) | <0.001 |  |
| **Race** | |  |  |  |  |  |  |
| White | | References |  |  | References |  |  |
| Black | | 1.343(1.125-1.485) | <0.001 |  | 1.344(1.207-1.496) | <0.001 |  |
| Others | | 1.126(0.960-1.321) | 0.144 |  | 1.201(1.020-1.414) | 0.028 |  |
| **Marital status** | |  |  |  |  |  |  |
| Married | | References |  |  | References |  |  |
| Unmarried | | 1.103(1.035-1.176) | 0.003 |  | 1.311(1.224-1.405) | <0.001 |  |
| **Histological type** | |  |  |  |  |  |  |
| NS | | References |  |  | References |  |  |
| MC | | 1.884(1.754-2.024) | <0.001 |  | 1.120(1.036-1.212) | 0.005 |  |
| LR | | 1.190(1.011-1.401) | 0.036 |  | 0.943(0.800-1.113) | 0.491 |  |
| LD | | 4.392(3.772-5.113) | <0.001 |  | 1.927(1.632-2.275) | <0.001 |  |
| **Ann Arbor stage** | |  |  |  |  |  |  |
| Early stage | | References |  |  | References |  |  |
| Advanced stage | | 2.263(2.123-2.412) | <0.001 |  | 1.644(1.527-1.768) | <0.001 |  |
| **Therapy** | |  |  |  |  |  |  |
| Combined modality | | References |  |  | References |  |  |
| RT only | | 1.206(1.072-1.357) | 0.002 |  | 1.151(1.009-1.378) | 0.034 |  |
| CT only | | 2.461(2.254-2.687) | <0.001 |  | 1.502(1.367-1.651) | <0.001 |  |
| No/unknown | | 2.568(2.288-2.882) | <0.001 |  | 1.600(1.419-1.804) | <0.001 |  |

SHR, subdistribution hazard ratio; NS, nodular sclerosis; MC, mixed cellularity; LR, lymphocyte-rich; LD, lymphocyte-depletion; RT, radiotherapy; CT, chemotherapy.

**Supplementary Table 3.** Univariable and multivariable analyses of ACD in patients with cHL in the Surveillance, Epidemiology, and End Results database: Cox proportional hazards model.

| **Variable** | **Univariate analysis** | |  | **Multivariate analysis** | |
| --- | --- | --- | --- | --- | --- |
|  | **HR (95%CI)** | **p-value** |  | **HR (95%CI)** | **p-value** |
| **Ages, year** |  |  |  |  |  |
| 20-39 (youngest) | Reference |  |  | Reference |  |
| 40-59 (older) | 2.577(2.424-2.740) | <0.001 |  | 2.602(2.442-2.773) | <0.001 |
| ≥60 (oldest) | 10.058(9.486-10.660) | <0.001 |  | 9.539(8.953-10.163) | <0.001 |
| **Period** |  |  |  |  |  |
| 1983-1992 |  |  |  |  |  |
| 1993-2000 | 0.813(0.767-0.863) | <0.001 |  | 0.808(0.761-0.859) | <0.001 |
| 2001-2005 | 0.739(0.696-0.786) | <0.001 |  | 0.714(0.669-0.761) | <0.001 |
| **Sex** |  |  |  |  |  |
| Male | Reference |  |  | Reference |  |
| Female | 0.699(0.666-0.733) | <0.001 |  | 0.726(0.691-0.762) | <0.001 |
| **Race** |  |  |  |  |  |
| White | Reference |  |  | Reference |  |
| Black | 1.215(1.124-1.314) | <0.001 |  | 1.251(1.155-1.354) | <0.001 |
| Others | 1.072(0.949-1.211) | 0.266 |  | 1.140(1.009-1.289) | 0.035 |
| **Marital status** |  |  |  |  |  |
| Married | Reference |  |  | Reference |  |
| Unmarried | 0.986(0.941-1.034) | 0.562 |  | 1.366(1.300-1.435) | <0.001 |
| **Histological type** |  |  |  |  |  |
| NS | Reference |  |  | Reference |  |
| MC | 2.160(2.051-2.276) | <0.001 |  | 1.157(1.095-1.222) | <0.001 |
| LR | 1.485(1.326-1.664) | <0.001 |  | 0.988(0.881-1.109) | 0.837 |
| LD | 4.301(3.837-4.821) | <0.001 |  | 1.813(1.613-2.038) | <0.001 |
| **Ann Arbor stage** |  |  |  |  |  |
| Early stage | Reference |  |  | Reference |  |
| Advanced stage | 1.809(1.726-1.896) | <0.001 |  | 1.409(1.337-1.484) | <0.001 |
| **Therapy** |  |  |  |  |  |
| Combined modality | Reference |  |  | Reference |  |
| RT only | 1.536(1.419-1.663) | <0.001 |  | 1.196(1.100-1.299) | <0.001 |
| CT only | 2.262(2.119-2.415) | <0.001 |  | 1.364(1.273-1.461) | <0.001 |
| NO/unknown | 2.468(2.269-2.685) | <0.001 |  | 1.604(1.472-1.747) | <0.001 |

ACD, all causes of deaths; cHL, classic Hodgkin lymphoma; HR, hazard ratio; NS, nodular sclerosis; MC, mixed cellularity; LR, lymphocyte-rich; LD, lymphocyte-depletion; RT, radiotherapy; CT, chemotherapy.
